# *“I just wanted to be like everyone else…”*: Qualitative exploration of women’s perspectives on female genital mutilation/cutting and its potential abandonment in Somalia

**DOI:** 10.1371/journal.pgph.0004571

**Published:** 2025-07-09

**Authors:** Zamzam I. A. Ali, Mervat Alhaffar, Natasha Howard

**Affiliations:** 1 Department of Global Health and Development, London School of Hygiene and Tropical Medicine, London, United Kingdom; 2 Center for Clinical and Translational Science, Mayo Clinic, Rochester, Minnesota, United States of America; 3 Syria Research Group (SyRG), co-hosted by the London School of Hygiene and Tropical Medicine and the National University of Singapore School of Public Health, United Kingdom & Singapore; 4 Saw Swee Hock School of Public Health, National University of Singapore and National University Health System, Singapore; PLOS: Public Library of Science, UNITED STATES OF AMERICA

## Abstract

Somalia has the highest estimated prevalence of female genital mutilation/cutting (FGM/C) globally. However, significant literature gaps exist on associated knowledge and contextual factors within the country, particularly among affected women. This study thus aimed to explore Somali women’s perspectives about FGM/C and its potential abandonment in Somalia. We conducted an exploratory qualitative study, informed by critical feminist and social norms theories. Data from 20 semi-structured remote interviews with women in Somalia were analysed thematically using abductive coding. We generated three themes of normative expectations and social control, patriarchal influence and gendered power, and FGM/C support and abandonment. Women demonstrated detailed knowledge of FGM/C procedures and consequences, sharing personal experiences of living with the effects of FGM/C on their physical, mental, and sexual health. Most supported continuation of “milder” ‘*gudniinka sunnah’* FGM/C, rather than abandoning the tradition. However, ‘*gudniinka sunnah*’ described several types of cutting, some which fit the World Health Organization typology of Type-IV (pricking, scraping) and others Type-I (removing part/all of the external clitoris), while ‘*gudniinka fircooniga’* (Type-III) – though still occurring - was universally described negatively. While acknowledging potential harms, women expressed the need for more religious and scientific clarity before changing their generally supportive opinions of ‘sunnah’ FGM/C. Discussing socio-cultural and religious reasons for this preference, women described social norms of femininity and how awareness interventions affected their understanding of ‘severe’ FGM/C and could help responses against FGM/C in Somalia. While all FGM/C types are negatively associated with women’s health and wellbeing, Type-IV is least understood. Although women opposed Type-III, they supported continuing Type-I/Type-IV as ‘sunnah’ FGM/C. Reasons for this practice are rooted deeply in Somali culture and require active engagement of a range of community stakeholders to enact meaningful changes. Health education can contribute to changing attitudes towards FGM/C in Somalia but is insufficient on its own, potentially benefitting from interventions to change social norms.

## Introduction

Female genital mutilation/cutting (FGM/C) is a harmful practice that contravenes the rights of women and girls [1,2]. Globally, approximately 200 million have undergone FGM/C in 30 countries with 3.6 million girls at risk annually [1]. This procedure is usually performed on young girls, and because it violates their right to health, physical integrity, freedom from torture and degrading treatment, and life - since it can lead to death [3], the universal declaration on human rights and other declarations and conventions maintain a zero-tolerance stance against FGM/C [4].

The World Health Organisation (WHO) classifies four FGM/C types: (i) Type-I clitoridectomy, partial or total removal of the clitoral glans; (ii) Type-II excision, partial or total removal of the clitoral glans and labia minora; (iii) Type-III, infibulation, the narrowing of the vaginal opening by cutting and stitching the labia minora and majora; and (iv) Type-IV, any other harmful procedures to female genitalia without medical reason [2,4]. All FGM/C types are associated with negative health consequences, commonly retention of urine, excessive bleeding, pain, genital tissue swelling, and problems in wound healing [5]. Longer-term, FGM/C can lead to recurrent vaginal infections, pelvic infections, menstrual cycle complications, obstetric complications (e.g., higher prevalence of obstructed labour, genital tearing, caesarean section, instrumental deliveries, haemorrhage, stillbirth, early neonatal death [5,6]), and psychological effects such as anxiety, depression, and post-traumatic stress disorder [4]. FGM/C is also associated with genitourinary and sexual problems such as genital tissue damage, frequent vaginal discharge and itching, painful intercourse, decreased/no sexual desire, decreased/no sexual satisfaction [5,6]. Type-III can additionally interfere with medical procedures such as gynaecological examination, cytology testing, insertion of intrauterine devices, and post-abortion evacuation procedures [6].

Somalia, an east African country experiencing over two decades of devastating civil conflict [7], has lacked a functioning central government since the 1991 collapse of the Somali state [8]. It faces economic challenges, with approximately 80% of Somalis under age 40 and the lowest rates of labour force participation in the region [7]. Somalia also has the highest estimated FGM/C prevalence globally at approximately 98% of girls and women aged 15–49 nationally, and highest in central and southern regions [9,10]. The average age at which FGM/C is performed is 6 years, varying across Somali regions [11]. Although FGM/C data in Somalia are extremely limited, WHO estimates 90% is Type-III [9]. There are no legal restrictions on FGM/C in Somalia, which encourages its continuation in the country and facilitates cross-border FGM/C for Somali women and girls in neighbouring and diaspora countries [10].

FGM/C is a complex social practice, deeply rooted in Somali cultural traditions. It is thus vital to foreground women’s voices, document their challenges and lived experiences, consider sociocultural sensitivities, and co-develop any policies or practices to reduce FGM/C in Somalia with affected women. However, data on women’s perspectives in Somalia are limited. Social norms theory, developed and expanded by scholars across sociology, psychology, and other disciplines, considers how cultural expectations influence behaviour [12,13]. Critical feminist theory highlights how patriarchy and power dynamics shape women’s agency [14–16]. These theoretical approaches enable interpretation of Somali women’s perspectives and experiences, considering socio-cultural realities and structural inequalities, to support the conceptualisation and design of interventions to contest potentially harmful norms while respecting cultural context.

We thus aimed to explore Somali women’s perspectives on FGM/C and its potential abandonment in Somalia, informed by social norms and critical feminist theories. Objectives were to: (i) explore women’s perspectives and experiences of FGM/C in their communities, including potential differences related to education or age; (ii) consider potential determinants of FGM/C continuation; and (iii) identify factors that could contribute to FGM/C abandonment in Somalia.

## Methodology

### Ethics statement

We obtained ethics approval from the Somali Research and Development Institute in Somalia (reference SORDI-EA0542) and the MSc research ethics committee at the London School of Hygiene & Tropical Medicine in the UK (reference 27217).

### Study design

In this qualitative mono-method study, we conducted remote audio-only semi-structured interviews with adult Somali women living in Mogadishu and Kismayo cities, the capital of Somalia and commercial capital of Jubaland region respectively [7]. We used critical feminist theory to guide our choices to: (i) use qualitative methodology to emphasise participant voices and lived experiences; (ii) use emancipatory axiology – valuing research as a social justice tool to better understand and improve people’s lives [17,18]; (iii) conduct heterogenous purposive sampling - to include intersecting identities and contexts: and (iv) consider reflexivity in data collection and analysis [19]; and as an interpretive lens to explore how patriarchy, gender norms, and hierarchical power relations might influence women’s discussions of FGM/C [20].

We used social norms theory in considering how FGM/C as practice was reinforced or challenged within Somali communities and how normative expectations and social pressures influenced women’s (and men’s) interactions, agency, and decision-making. While acknowledging its scholarly criticisms, such as reductionism and insufficiency, we considered it potentially useful in dialogue with critical feminism, in considering potential influencers, private oppositions, and collective behaviours [12,13]. We drew particularly from Bicchieri’s approach (2006), which emphasises social norms in shaping individual behaviour and facilitating social coordination and order [12]. Bicchieri conceptualised norms as: (i) *shared expectations* about appropriate behaviour, guiding individuals by defining acceptability; (ii) *descriptive or injunctive,* distinguishing descriptive as perceptions of what others actually do and injunctive as perceptions of what others (dis)approve of, with injunctive norms potentially more influential in decision-making; (iii) *conditional preferences*, suggesting individual behaviour is influenced not just by personal preferences but also by expectations about and conformity with others’ behaviour; (iv) *norm compliance and enforcement,* ensured though social disapproval or punishment of norm violators and social approval/rewards for norm followers, reinforcing adherence; and (v) *norm formation and change*, with *formation* and *maintenance* through norm emergence – in which individual behaviours are shaped by observations of others combined with perceived social (dis)approval, and *change* through processes of internalising social influencing and learning [12].

Our research question was intentionally broad: *“How do Somali women perceive FGM/C and efforts to support its abandonment in Somalia?”*

### Sampling and recruitment

We used heterogeneous purposive sampling and snowballing to identify and recruit participants of different ages and educational backgrounds, from an initial list of 8 of ZA’s professional contacts. Eligible participants were Somali women, 18 + years old, living in Mogadishu or Kismayo cities in 2022. To increase diversity, we purposively differentiated by age (i.e., younger at 18–30 years, older at 31 + years) and education level (i.e., none, Dogsi/primary/secondary, higher education). In Somalia, students attend religious schools (i.e., Dogsi) prior to starting formal education (e.g., learning Quran, reading, writing), while higher education included those who attended university or postgraduate studies. One eligible participant refused due to discomfort talking about FGM/C. We excluded women living in displacement camps or rural areas with poor internet due to technical limitations, while recognising this potentially limited our sample.

ZA shared the study information sheet and consent form - developed in English and translated into Somali - with potential participants, meeting each remotely via WhatsApp or Zoom applications to explain they were free to stop anytime, how their privacy and confidentiality would be ensured, and address any concerns. ZA recorded verbal informed consent for all participants prior to interview, as only five could provide written consent due to unreliable e-signatures/printing access.

### Data collection

We developed the semi-structured interview guide in English and translated it into Somali, the native language of interviewer and participants. It included three sections on: (i) participant characteristics, i.e., age, education, occupation, and marital status; (ii) knowledge and experiences of FGM/C; and (iii) determinants of FGM/C continuation at family, community, and societal levels (e.g., culture/traditions, religion, education, legislation/policy, politics). The guide was sufficiently flexible to enable inductive probing, allowing ZA to clarify meaning and participants to expand on perspectives, stories, and experiences.

ZA collected data in June-July 2022 in Somali or English using Zoom (6 interviews) and WhatsApp (14 interviews) applications, depending on participant preferences. All except SW1 were conducted in Somali by participant preference. As FGM/C was potentially sensitive, privacy and confidentiality were supported by enabling participants to stop at any time or avoid uncomfortable questions, and scheduling interviews according to participant preferences (e.g., some as early as 04:00 UK time or late at night after children were asleep for greater privacy). Interviews were conducted until we deemed data saturation achieved (i.e., no new information from participants, as proposed by Glaser and Strauss [21]). Interviews took an average of 45 minutes and were audio-recorded, translated, and transcribed in English by ZA. We stored audio files, transcripts, and field notes (after redacting all personally identifiable information and assigning anonymised identification codes) on a secure institutional server only accessible to the research team.

### Analysis

We analysed data thematically in NVivo-12, using a reflexive abductive approach adapted from Braun & Clarke [22]. This included data familiarisation; generating initial codes; combining codes into initial themes; abductively moving back and forth between data and theoretical concepts to guide interpretation (i.e., engaging in a dialectical process of abduction, deduction, and induction, with theory informing analysis and data informing interpretation); (re)naming themes, and selecting quotes that exemplified participant voices to represent themes and sub-themes.

### Reflexivity

ZA, a female Somali clinician - working in Kismayo since 2019 but born and raised in Saudi Arabia – conducted data collection and initial analysis. Having spoken Somali since childhood, her positionality is partially local but primarily of the diaspora, with participants responding to her as a compromise of local and foreigner. Her gaze is informed by FGM/C perceptions shaped by her mother and the Somali community in Saudi - who supported milder forms as a perceived religious requirement - while acknowledging the strong influences of Saudi diaspora upbringing, medical training, involvement in an anti-FGM/C campaign in Sudan (Salema), and negative global normative framing of FGM/C. MA and NH are experienced qualitative researchers, focused on public health and health equity issues in the Arab and Muslim world, and based in London and Singapore respectively.

### Patient and public involvement

Patients or the public were not involved in the design, conduct, reporting, or dissemination planning of our research.

## Findings

### Participant characteristics

Table 1 shows sociodemographic characteristics of 20 participants. A heterogeneous sample of ages (i.e., 12 younger [averaging 22 years], 8 older [averaging 44 years]; total average age 31, ranging 18–80 years), education (i.e., 6 no formal education, 7 Dogsi to secondary, 7 some higher education), marital status (i.e., 11 unmarried, 8 married, 1 divorced), residency (7 Kismayo, 13 Mogadishu), and socioeconomic status (i.e., 10 homemakers/no paid employment, 1 student, 9 working externally in various professions) encouraged diversity of perspectives.

**Table 1:** Participant characteristics.

ID	Age	Marital status	Education	Occupation
SW01^a^	26	Single	University	Public administrator
SW02	31	Single	University	Doctor
SW03	27	Married	University	Psychologist
SW04	21	Married	University	Nurse
SW05	29	Single	University	Charity staff
SW06	18	Single	Dogsi-Secondary	Student
SW07	22	Single	University	Nurse-Midwife
SW08	50	Married	Dogsi-Secondary	Homemaker
SW09	43	Married	Dogsi-Secondary	Business owner
SW10	48	Married	Dogsi-Secondary	Homemaker
SW11	33	Married	No formal education	Cleaner
SW12	21	Single	Dogsi-Secondary	None
SW13	20	Single	No formal education	None
SW14	19	Single	No formal education	None
SW15	21	Single	No formal education	None
SW16	19	Single	No formal education	None
SW17	77	Divorced	No formal education	None
SW18	36	Married	Dogsi-Secondary	Homemaker
SW19	26	Married	Dogsi-Secondary	Homemaker
SW20	35	Single	University	M&E officer

NB: conducted in English in accordance with participant’s preference.

### Thematic findings

We generated three themes: (i) normative expectations and social control; (ii) patriarchal influence and gendered power; and (iii) FGM/C support and abandonment, under which we reported related subthemes.

### Normative expectations and social control

This theme, primarily generated in dialogue with Bicchieri’s social norms theory (2006), explored what women considered acceptable regarding FGM/C within their communities and how social expectations shaped related attitudes and decisions [12]. It included three sub-themes: (i) defining and typologising FGM/C; (ii) FGM/C procedures; and (iii) belonging and social control.

#### Defining and typologising FGM/C.

Participants used “*gudniinka*,” meaning circumcision, as the Somali word for FGM/C. Participants were familiar with FGM/C and framed it normatively, rarely pausing to think before responding. Most mentioned learning about FGM/C primarily through experiencing it, secondarily from older family or community member expectations, and thirdly a few mentioned school and Dogsi.

*“I heard and learned this first [because] I, who am talking to you now, was circumcised wasn’t I? My siblings were circumcised. All our people knew it this way and this was their system, wasn’t it?” (SW10)*

Many also described FGM/C as a defining stage in a girl’s life and a fate every girl experienced, highlighting its descriptive normativity.

*“FGM/C is a stage all girls go through.” (SW6)*

A minority, with no education, denied any knowledge of FGM/C and appeared uncomfortable discussing it, suggesting injunctive assumptions about talking about sensitive issues with a stranger not seen among more educated women.

*“No, no, no, I didn’t learn about FGM/C or things like that. I don’t know these things.” (SW11)*

Many defined FGM/C as mutilation of female genitalia, predominantly describing Type-III, in which all or part of the external genitalia are removed and the vaginal opening narrowed.

*“What I understand from FGM/C is it’s mutilation of the female reproductive system, mainly the labia majora and basically the clitoris” (SW5)*

All participants typologised FGM/C as either ‘*gudniinka fircooniga*’ (i.e., ‘pharaonic circumcision,’ equivalent to Type III) or ‘*gudniinka sunnah*’ (i.e., religiously sanctioned or ‘good’ circumcision). However, what different participants defined as ‘sunnah’ varied between describing Type-I (removing part/all the external clitoris) or Type-IV (pricking, nicking).

*“*The Sunnah FGM, which is not - you know it doesn’t cause a lot of harm - where they just remove the clitoris*”* SW5 (suggesting Type-I)

“*The sunnah circumcision where the girl will bleed a little bit*...” SW7 (suggesting Type-IV)

While some women detailed a range of interpretations, further analysis showed no clear definition for ‘*gudniinka sunnah*’ and that we could not typologise it neatly as Type-I, as we had initially understood, nor was it universally a prick/cut with no flesh removed.

“*Sunnah type is two options. Some people say suture it one suture, so they cut a little from the tip of the clitoris, and after they cut the frontal tip they suture it 3 or 4 sutures, and some people only cut the front tip - to bleed only - they only bleed the tip.., no sutures will be done*.” SW20

“*They cut the clitoris, it’s lower part, and after that cut the sides [labia], and sometimes they cut the clitoris only completely and they leave the sides, and sometimes they even leave parts of the clitoris and only cut its tip. Each people do differently... these are the three types I saw*.” SW10

Some participant accounts indicated potential shifts due to urban medicalisation, with midwives preferring to inflict a prick to allow at least one drop of blood, while traditional practitioners might cut more deeply. However, this could not be confirmed, and further research is needed.

Participants often used ‘good’ or ‘bad’ circumcision to describe ‘*sunnah’* or ‘*fircooniga’* (pharaonic) respectively. Thus, pharaonic FGM/C was frequently identified as ‘bad or wrong’ and sunnah as good, acceptable, or the ‘right type of circumcision.’ For example, a nurse typologised normatively though she deviated in describing both negatively.

*“One, as the mothers call it to make it a good thing, they call Sunnah circumcision, and one they call Ferron [Pharaonic], the bad circumcision, very bad. So, they divide it into these two types and mainly they say the girls will be circumcised the Sunnah type, the good one. But none of it is good…” (SW4)*

While most participants suggested that only Type-III was harmful, one participant described all FGM/C as ‘an assault.’

*“Sometimes there are situations where girls are assaulted and harmed very much by completely removing their important and beneficial organ.” (SW1).*

#### FGM/C procedures.

Participants explained the processes of conducting both Pharaonic and Sunnah FGM/C in urban and rural areas, as women usually gather when FGM/C is performed, and many had observed FGM/C done to their daughters, sisters, or neighbours. As we could only include urban women due to internet limitations, we relied on those women previously living rurally to understand major urban-rural differences. A few discussed pre-FGM/C processes, and how families prepared girls with gifts - thus rewarding social compliance.

*“What they do is before they circumcise girls, they give them gifts so that they won’t be shocked and to please them. So, they took the girl and bought gifts for her. Even me, I felt happy for her and said circumcise me too so that I get these gifts.” (SW4)*

Some participants highlighted urban/rural differences, such as medicalised Type-I and Type-IV predominating in urban areas. Urban families used midwives, considered better trained and more knowledgeable in avoiding complications - and who only perform ‘*gudniinka sunnah*’ - thus normalising this in cities. In contrast, rural families relied on traditional cutters and were reportedly less aware of FGM/C types or extent of cutting. Thus, some participants who previously lived rurally described excessive bleeding and the need for herbal treatments as common. Several participants explained FGM/C medicalisation in cities.

*“The girl is cut from the tip of her clitoris. First, they inject it with an anaesthesia injection. I was standing there when all my girls were circumcised. The anaesthesia is injected into both sides, from the tip of the clitoris, ‘shruug’ [imitating the sound of injected fluid]. Then the needle is inserted in the other side. All sensation is gone. After that, they take the knife [scalpel] and hold it like that. Then it is cut, ‘gurduf’ [imitating the sound of cutting], and after that it’s sutured.” (SW8)*

Older participants similarly explained how FGM/C was done traditionally and in rural areas, using tools from the environment such as thorns, herbs to stop bleeding, and boiled water for disinfection.

*“In the past? There weren’t scissors, no knives [scalpel], there was only a small knife done by craftsmen, a small one wrapped in a small piece of cloth. Girls were circumcised by it and after they were cut completely, some people put thorns then put a grain of millet in the opening, if it is larger than the grain it is a shame to the girl” (SW17)*

Rural participants explained the post-FGM/C period, and the difficulties they saw girls experiencing.

*“I saw many girls who have been cut repeatedly then sutured multiple times, then for more than ten days being lifted up and down by someone else* [requiring assistance to move]. *A lot of suffering. I even saw girls that when they are circumcised and sutured can’t find a way to urinate because of all the Malmal [Myrrh leaves] that was applied after cutting to make it attached. So, they recall the women [circumciser] and she cut again and cause another wound.” (SW19)*

#### Belonging and social control.

Many participants described FGM/C in terms that related to belonging and the social control or coercion this engendered. Most suggested that FGM/C persisted because of its normative value in Somalia, as inherited culture across generations that could not easily be abandoned.

*“Somali people are very attached to their culture, and this circumcision originally came from the past. I mean from ancient times till now. It’s still accompanying us, and it became part of the culture, even an essential part.” (SW6)*

They described FGM/C supportive culture across communities, including the political community.

*“The whole culture in Somalia, whether it’s in politics or government, they still believe in FGM/C. It doesn’t matter where the person is, it can be the government, it can be the community, they mostly believe the same thing.” (SW5)*

Some framed FGM/C as Somali pride, describing how important it is for them as mothers to cut their daughters to maintain their pride as women and improve their daughters’ honour and reputation.

*“Why wouldn’t I circumcise her? It is something in our hearts, all of us. It is a compulsory thing in our hearts. If we don’t circumcise our kids, we would be embarrassed by them. Even when we were young, we used to be proud of it.” (SW9)*

*“We went through this* [FGM/C]*, and our girls will take the same path.” (SW1)*

Many highlighted social pressure as strong motivation to continue FGM/C, describing how parents of uncircumcised girls could face coercion, particularly from older family and community members, influencing their decisions about FGM/C for daughters regardless of knowledge about its harms.

*“There are many people who are aware of the harm of FGM/C, but they say what could we do if my parents are resisting it [FGM/C abandonment]. So, the grandparents will use things like ‘I will curse your kids!’ or ‘Do what I want!’.” (SW3)*

*“A lot of questions people ask, intensively, what are you going to do? Will you leave them like this? Or are you planning to do it? What’s the matter with you? Why haven’t you done it yet? Why haven’t you circumcised your girls yet?” (SW18)*

Social coercion included discrimination against uncircumcised girls through gossip, insults, and community exclusion.

*“I just wanted to be like everyone else, like just not to feel like I’m the odd one out.” (SW5)*

*“I am against not touching girls [FGM/C; nervous laugh] because in Somalia they will insult the girl, saying that she has extra edges* [clitoris or labial skin]*. If the girl hasn’t been touched* [circumcised]*, they will assault her* [insult her honour] *and say she still has the clitoris…” (SW8)*

Some suggested further that mothers supported FGM/C to protect their daughters from sexual desires and prevent unwanted extramarital relations with men. This was expressed among all age groups and educational backgrounds.

*“People might say, if you don’t circumcise the girl, she’s going to be wild…” (SW5)*

*“Mothers, even if they are educated, might still circumcise their girls and the reason behind that they might believe this is a girl, if I don’t do this, she might fall in any mistakes [extra-marital relations]. That it will be easy for her to do so because they believe her [sexual] desire will disappear if they cut her and she will not look for a man or want a man.” (SW3)*

Less frequently mentioned reasons aligned with concepts of general purity, cleanliness, and beauty.

*“Parents would say X or Y fixed their daughter and made her more beautiful so why don’t we do that?” (SW18)*

*“By doing so they believe they are benefiting the girl and if they don’t cut and suture girls then they won’t be clean.” (SW3)*

### Patriarchal influence and gendered power

This theme explored patriarchy, power, hegemonic masculinity, coercion, and opportunity in shaping FGM/C’s normative value in Somali communities. It included three subthemes: (i) male support and ignorance of FGM/C; (ii) marriageability; and (iii) intimacy preferences.

#### Male support and ignorance of FGM/C.

Many participants identified men as powerful actors in ongoing FGM/C practices, reflecting on men’s (in)direct positionality in FGM/C decision-making through perceived preferences for circumcised wives and insufficient interest in its harms. Many comments indicated men’s ignorance and lack of engagement in FGM/C decision-making, describing mothers as the driving force behind FGM/C in families.

*“Men didn’t care about women, because if they cared, if fathers who know religion, as the house leader, he should say no one will do this [FGM/C] to my daughter.” (SW3)*

However, when involved, some shared how fathers’ opinion about FGM/C was powerful and could modify women’s decisions.

*“My father, when we were about to be circumcised, told my mother ‘If you do the Ferron circumcision to my daughter, I will not forgive you in this life or the next* [afterlife]*.’ So, we as a family are not supporters of FGM/C.” (SW1)*

*“There was a girl who recently came from Saudi - old girl who is 11 years old. Her father said she must be circumcised. I said this is an old girl. It is shameful to circumcise her at this age, but he refused. He said ‘It is a must. She will be circumcised!’” (SW17)*

Some university-educated women discussed their hoped-for husbands’ support in overcoming social pressures if they did not want to circumcise their daughters.

*“It’s a conversation I’m willing to have with my partner before we even start a family. I want to have that conversation, so we don’t clash, and he knows that, you know, that we don’t plan on circumcising our daughters if we have daughters.” (SW5)*

#### Marriageability.

Marriage was a strong motivation for FGM/C, with several participants linking FGM/C to marriageability within the Somali community and implicit requirements by men or their families before marriage. This was often articulated normatively in terms of sexual purity and cleanliness, with men preferring to marry circumcised girls to ensure their virginity. It is important to clarify that ‘girl’ in this context referred to social status as a virginal unmarried female rather than a specific age range (akin to English terms ‘maiden’ or ‘damsel’), while also used to mean female child depending on context.

*“Somali men, some of them believe that if the girl is not circumcised then she is not a ‘girl’* [virgin]*. Especially if she is not circumcised the Ferron type, then they might not believe she is worth marrying and establishing a family with. So, for example, if you ask a mother ‘Why are you circumcising your girl? Why don’t you let her avoid the problems of FGM/C?’ She will say ‘And who will marry my girl?’” (SW1)*

*“If a man was given a girl [married to an uncircumcised maiden], he will say ‘She is not a girl [virgin]...’ He thinks it* [the vagina] *is this area that is completely closed and needs to be reopened* [referring to Type-III FGM/C]*. So, he thinks this is the right way. It [sexual intercourse] is difficult for him and it is difficult for her, but he says ‘I want it this way.’” (SW17)*

Some described how men could question and ruin the reputation of uncircumcised women, making families afraid to stop practising FGM/C as it remained an important demonstration of girls’ virginity and therefore respectability and social value.

*“Showing that the girl is still a girl* [virgin] *when she is being married to a man, then she will not be returned with a scandal* [accusations of immorality…]*. In the past, it was a disgrace if a girl is not circumcised and the man when he marries your girl, they say if the girl is not a girl they can tell from some signs; for example, that she has been used and her virginity is incomplete if she is not sutured. So, because of this people had to do it.” (SW8)*

#### Intimacy preferences.

Participants shared how FGM/C affected their intimate relationships, describing normative challenges in speaking about intimacy. In describing sexual difficulties caused by FGM/C, several noted that men might avoid taking their wives to doctors for such issues, indicating masculinist assumptions that men should not require assistance to have sexual relations with their wives could threaten their manhood and reputation.

*“Sometimes it can happen that this girl is taken to a doctor and they say ‘Let us cut her* [de-infibulation]*’ and if this is done it will bring shame on the man [husband] because they will say ‘He is a man and he couldn’t even open the women* [failed sexual penetration]*!’ So, problems start from there for her. She will be forced to bear this hardship and suffering, and she continues like that* [to endure painful sex]*, or they go to a doctor and reopen her. And if she was taken to a doctor the man will not accept. He will not accept. They say culturally, or as a Somali norm, she was reopened for him* [he was so weak he needed assistance]*. Umm, it is a problem because they will say he is not even a man. There is something wrong with him.” (SW20)*

Several also suggested (counter-hegemonically) that FGM/C affected men negatively by diminishing their sexual satisfaction, especially because some women experienced limited/no sexual attraction to men due to FGM/C. They suggested men increasingly preferred marrying uncircumcised women to avoid risking their intimate relationship with their wives.

*“When she marries, a lot of troubles and suffering she will face to the extent they both go to the hospital and her husband says, ‘My wife and I are together for this long and still couldn’t do anything* [have sex]*.’” (SW18)*

*“There are a lot of married couples who have problems in bed. The man would say ‘My wife doesn’t have any sexual desire in me and she doesn’t care about this,’ and many people in their mind might think all women are like this and they shouldn’t have any sexual desire, and some women even say the girl shouldn’t have any sexual desire. This is wrong.” (SW3)*

Alternatively, a participant shared her perception that Somali men may prefer Type-III FGM/C, as this challenging sexual intercourse demonstrated his manhood and the woman’s purity.

*“Some Somali men believe to circumcise the girl very well and tighten her, which is to, umm, to circumcise her very well* [Type III] *so that this man will have difficulty with her* [difficult sex]*. This is what they say he wants, to have a difficult time with her. If she is Sunnah circumcised, how would he know if she is virgin or not?” (SW20)*

### FGM/C support and abandonment

This theme highlighted both complexities and opportunities for agency, resistance, and social change: (i) framing FGM/C support and ‘Sunnah’ preferences; (ii) religious interpretations; and (iii) reflections on FGM/C abandonment.

#### Framing FGM/C support and ‘Sunnah’ preferences.

All participants supported abandonment of ‘Pharaonic’ (Type III) FGM/C due to its many harms.

*“I would advise the Somali people that circumcision has different types. For the one they just bleed it is fine, but there is one where the part of girls, their femininity…their womanhood and motherhood would be attacked, and she will suffer in that type, the Pharaonic, so I would say let us stop it.” (SW6)*

Some also expressed regret about going through Pharaonic FGM/C and living with its consequences.

*“If I knew in the past it is like this, I would educate others, and we might not even be circumcised. We wouldn’t suffer from these troubles [...]. We wouldn’t go through any of this, my daughter, if we knew, and if we had the mindset we have now and how we understand it.” (SW8)*

Some framed Type-III FGM/C as ‘unnecessary,’ with harmful long-term implications for women’s wellbeing, suggesting potential scope for abandonment.

*“I would like them to understand that the girl will not gain anything from it except pain. I mean persistent pain that will always be with her. The place that is mutilated, and it is not reversible, you can’t correct that. Forever she will live with it. The girl will feel incomplete, unequal, all the time […] I would like them* [Somalis] *to know this.” (SW2)*

However, most participants supported continuation of *‘gudniinka sunnah*’ (Type-I or Type-IV FGM/C), particularly non-educated and moderately educated women, justifying it as normal and ‘no problem’ as it was associated with fewer medical complications than Type-III. Many comments, such as the following, suggested it was ‘just a prick’ while other descriptions indicated it involved more extensive cutting, though less than Type-III.

*“The Sunnah you hear is not something problematic. It is just bleeding the girl a little bit. No problem in that. I would circumcise her the Sunnah type.” (SW13)*

While we could not interview midwives or traditional cutters to confirm severity, women themselves described a range of cutting – from a prick and blood drop to clitoral removal - as *‘gudniinka sunnah*.’

#### Religious interpretations.

While all participants framed FGM/C in religious terms, interpreting it through Islamic teachings and requirements regardless of educational background, opinions diverged strongly. Some – particularly university-educated women - indicated FGM/C was antithetical to Islamic beliefs, not a religious requirement, and should be abandoned.

*“I can support what my religion tells us is mandatory and right, […] but this is not in my religion. It is a bad thing from my culture that harms people” (SW4)*

Most university-educated participants described FGM/C negatively, articulating a globally recognised zero-tolerance stance against all types of FGM/C drawing from religious conceptualisations.

*“I don’t support it at all because you know Allah made us whole [..]. It’s not a mistake and we deserve to be whole and so I don’t support it at all. I, if I have girls Inshallah, I won’t circumcise them […]. It hasn’t caused a health issue for me but at the same time, you know, it’s really unnecessary because it doesn’t change your personality. It doesn’t make you be something you’re not.” (SW5)*

Others – particularly those with less formal education - used Islam to justify FGM/C and linked it to religious identity, describing it as religious obligation, particularly *‘gudniinka sunnah*.’

*“My opinion of circumcision is like the opinion of our religious leaders. They say do the Sunnah” (SW3)*

*“So, if you can’t circumcise or suture her, it is a must to at least bleed her a little as the religion states” (SW14)*

Several suggested Somali mothers had insufficient religious knowledge and were prone to following religious misinformation about FGM/C, acknowledging the influence of (male) religious leaders in shaping community understanding and beliefs about FGM/C.

*“The problem in our community, women are troubled because women don’t have enough religious knowledge. It didn’t reach them well, and no religious leaders took the responsibility of teaching them. The religious leaders didn’t try to teach women ‘Don’t do this [FGM/C]! It is forbidden.’” (SW3)*

#### Reflections on FGM/C abandonment.

In considering opportunities to abandon FGM/C in Somalia, participants described changing trends. Some suggested shifting community norms with growing realisation of FGM/C harms.

*“Alhamdulillah [praise God], now people understand more, and no one does it. It seems like it will end soon.” (SW8)*

Many expressed optimism about ending FGM/C, especially older participants who witnessed changing community norms and geographic differences.

*“In the past, many Somalis did it, but recently people lost interest in it, and they are becoming better. Not like in the past. People started to understand and change.” (SW15)*

*“Some people in some districts don’t accept [change]. In the Northern regions and Galmudug and around there it is a shame, and in Galkacayo it is a shame, but the Banadri [Mogadishu] and the Southern they cut small tip only. So now circumcision is better.” (SW17)*

Many participants described the impact of education in changing FGM/C norms in Somalia, with awareness interventions encouraging these trends.

*“The idea can spread between people, just like Corona [COVID-19] and how people infected each other. Ideas can be taken the same way.” (SW12)*

They supported this by suggesting isolation and insufficient knowledge explained higher pharaonic FGM/C rates in rural areas, though how much this was due to social ‘othering’ was unclear.

*“It was due to ignorance. No parent wishes to harm their children, it was what they believed in.” (SW12)*

*“They don’t have sufficient knowledge about this circumcision. It is due to ignorance. If they had enough details, if they knew the harm it causes, that it is playing with human sensations* [pleasure]*, they wouldn’t do it.” (SW13)*

Most participants were against legally restricting FGM/C in Somalia, suggesting people would continue FGM/C secretly and community engagement and awareness would become more difficult.

*“I can still hide and do it. If you make a law, then people can do it secretly. It is not important whether it’s illegal. You will not necessarily know who will do this bad thing [FGM/C]. Instead, what can be better - before considering legality - it is better if you first teach the person and make them aware.” (SW7)*

## Discussion

### Key findings

This exploratory study is an initial effort to understand women’s perspectives on continued FGM/C in Somalia and its potential abandonment. Findings add to the literature on FGM/C determinants and abandonment, showing more highly-educated participants were not automatically against FGM/C. Rather, we found that older women were more willing to abandon FGM/C, based on their own experiences and observations, while younger women were inclined to maintain traditions. Further research is needed to clarify this. Findings also contribute nuance to Somali definitions versus WHO typologies, with women referring to bad ‘*fircooniga’* and good ‘*sunnah’* circumcision (‘*gudniinka’)* though what constituted ‘sunnah’ differed. Lastly, the common conflation of FGM/C as religious requirement in Islam, which could only be abandoned if religious leadership confirmed this could be done, reinforces the literature on the importance of engaging religious leaders. It also suggests further religio-historical scholarship on FGM/C is needed beyond assurances that the practice is not required in any religious text [23,24].

FGM/C was described as deeply rooted in Somali culture and practised openly, with participants largely supporting continuation of ‘*gudniinka sunnah*’ (without always distinguishing between Type-I or Type-IV) for religio-cultural reasons ranging from presumed religious obligation, to maintaining cultural identity/pride, to ensuring their daughter’s future through adhering to standards of good girlhood (i.e., chaste, pure, clean, beautiful) to ensure honour, reputation, and thus a bright future through a good marriage. This aligns with the literature on FGM/C and Somalia [11] and the Africa region generally [25,26].

Interestingly, most supported abandonment of Type-III as harmful and unnecessary but wanted ‘*gudniinka sunnah*’ continued as ‘harmless bleeding’ that could maintain many of FGM/C’s perceived benefits. As descriptions of ‘*gudniinka sunnah’* varied between Type-IV (pricking) and Type-I (partial or total removal of the clitoral glans), it potentially includes more than ‘a bit of bleeding’ suggesting some participants minimised its potential harms as either type was less damaging than the commonly practiced Type-III. However, this preference did indicate potential scope for working with community leaders and influencers to urgently abandon the most harmful Type-III given the need for community ownership of any normative changes and long-term lack of success (and ethical concerns) with global ‘zero-tolerance’ approaches [27–29]. This supports findings in Somaliland - that 95% preferred Sunnah FGM/C for their daughters [11], and on changing FGM/C attitudes among Somali women in Norway - who similarly preferred ‘milder FGM/C,’ suggesting desire to retain cultural traditions in less debilitating ways [30].

Considering FGM/C as a social norm—a customary practice ingrained in cultural traditions and institutions—with adherence reinforced through social control, conformity, and socialisation, means interpreting many participants’ positive perceptions as potentially stemming from desire to conform to social expectations and avoid social sanctions associated with deviation for themselves or their daughters [31,32]. Positive FGM/C perceptions could be perpetuated through collective processes of intergenerational social influence, whereby women internalised normative beliefs and behaviours that FGM/C marks social identity and belonging, reinforcing felt cultural affiliation and community. Normative FGM/C behaviours were further reinforced through social rewards, such as respectability, belonging, and marriageability. Participants who identified FGM/C as a religious requirement appeared most strongly supportive of its continuance, aligning with findings that participants who linked FGM/C to their faith were less open to change [4]. Somalis primarily follow the Shafi’i school of Sunni Islam, which traditionally considers the practice obligatory [23,33]. Thus, many people – particularly rural/less educated – are unlikely to change their views unless religious leaders change their interpretation [26,34].

Considering FGM/C from a critical feminist perspective, participant’s ongoing support of FGM/C could be internalised patriarchal norms or acceptance of gendered power within communities [35]. Our participants described men as encouraging FGM/C as a prerequisite for marriage and proof of virginity, which aligns with survey results that 96% of Somali men preferred marrying circumcised women [11]. Despite recognising its harmful effects on women’s bodies and wellbeing, they could prioritise male control over female sexuality and reproduction with FGM/C described as a means of ensuring women’s chastity, purity, and marriageability and aligning with traditional gender roles supporting male dominance [36]. Thus, women’s support of FGM/C could be reinforced by socialisation processes perpetuating harmful gender stereotypes and practices of bodily control of girls and women. Women may internalise beliefs around FGM/C as necessary or a desirable ‘rite of passage,’ symbolising cultural identity and belonging, despite its adverse consequences [26]. As most women had undergone FGM/C, cognitive dissonance or psychological defences could help them rationalise FGM/C as positive to cope and justify it as maintaining cultural continuity and community belonging. Participants’ potentially contradictory perspectives on FGM/C could thus be seen as underscoring the complex ways gendered power and patriarchal norms shape women’s agency, identities, and experiences [36].

In actuality, both gendered power structures and social expectations likely mutually reinforce the practice by regulating women’s *and* men’s identities. For example, men’s support of FGM/C is often framed as maintaining patriarchal power, but social norms of masculinity also contribute. Thus, Somali men may support FGM/C because of expectations around women’s purity and marriageability and also expectations of their own sexual and social status (e.g., that a man must “open” an infibulated woman through penetration and failing to do so undermines his masculinity). This would suggest that FGM/C is sustained by patriarchal social norms that regulate both women’s bodies and men’s identities and therefore status [37]. As we did not interview men, further research on this is needed.

### Implications for abandonment

Implications for socio-political advocacy to abandon FGM/C or mitigate its harms, include women’s expressed interest in abandoning Type-III ‘pharaonic’ FGM/C, men’s perceived influence and desires within a patriarchal society, and contradictory (or internationally-informed) interpretations of religious guidance.

Using social norms theory to encourage abandonment requires shifting normative expectations through targeted interventions promoting alternative narratives, support networks, or community engagement initiatives fostering critical reflection, discussion, and collective action [38,39]. Engagement with religious leaders is essential to correct misconceptions about Sunnah FGM/C being recommended by Islam, which motivated many women to continue the practice [33]. Community engagement in developing and implementing any intervention could include forming an FGM/C strategy, action plan, and local authority FGM/C taskforce with representation of women, religious scholars, academics, and government with collaboration between different governmental ministries, including health and education, to add FGM/C education materials to the school curriculum.

Additional contributions of critical feminism to abandonment initiatives include the necessity of addressing underlying gender inequalities and promoting alternative narratives of masculinity and femininity to potentially include egalitarianism, bodily autonomy, sexual rights, and marital sexual enjoyment [40]. Related to this, education appeared to influence perspectives, with women with no or limited education appearing more supportive than those with post-secondary education, aligning with review findings on education to counter FGM/C in Africa [4]. However, awareness raising is unlikely to be sufficient alone as all expressed awareness of Type-III harms but preferred continuing ‘milder’ versions over abandonment. This mirrors Duncan and Hernlund’s description of awareness campaigns increasing awareness but not changing behaviour [41]. It is also worth noting, as found in other FGM/C practicing countries, that an overly simplistic zero-tolerance approach to FGM/C can be seen as an attempt to force Western values on practicing communities and attack cultural beliefs, identities, and religion, resulting in counterproductive defensive reactions [33,36]. Age also appeared to influence perspectives, with older women more willing to abandon FGM/C entirely, based on lived experiences – often of Type-III - on their health and wellbeing, while younger women appeared more dismissive of potential harms. This differs from Alo and Gbadebo’s conclusion that older people were more supportive due to increased youth education [4,42]. Further research is needed to understand this.

Further research is thus needed on the potential effects of education and age, such as through collaboration between health and women and human rights development ministries. For example, we did not ask about Somali clinician’s knowledge about FGM/C reconstructive surgeries, but an obstetrician’s description of FGM/C as ‘irreversible’ indicated further research on Somali doctors’ knowledge of reconstructive surgery may be warranted. While some participants had moved from rural areas and could provide insight on rural-urban differences in FGM/C practices, further field research in rural areas and displacement camps would be useful to overcome internet connectivity issues and further explore potential differences in FGM/C perspectives and practices. It would also be important to examine the prevalence of medicalised FGM/C and how this has affected health risks and perspectives in Somalia.

### Limitations

Several limitations should be considered. First, ZA conducted interviews remotely by audio call, which could affect rapport and responses in positive and negative ways, as noted by Douedari et al [43]. Video calls were not feasible given internet constraints. We also noted that participants appeared more comfortable with audio calls. As audio did not allow observation of expressions and body language, ZA paused to check in after sensitive questions, offered breaks, and monitored participants tone to ensure they were doing sufficiently well throughout. Second, to ensure internet access, our reach was limited to urban residents who may have been somewhat wealthier and more globally aware, though we made efforts to include a range of perspectives through our sampling strategy. As we excluded women in displacement camps and rural areas, further in-person research could explore potential differences in perspectives. Given the richness of responses and alignment with other literature these issues did not appear unduly detrimental. Third, we were unable to sufficiently explore intersections of age, education, and ethnicity due to time and resource constraints, nor did we try to include perspectives of men, religious leaders, or politicians though they are vital influencers. Future research should address these gaps.

## Conclusion

FGM/C is a Somali tradition, continuing through years of anti-FGM/C campaigning. This study, exploring Somali women’s perspectives on FGM/C and potential abandonment, found women rejected Type-III as harmful but supported continuing ‘milder’ (e.g., Type-I, Type-IV) Sunnah FGM/C for religio-cultural reasons. Health education can contribute to changing attitudes towards FGM/C in Somalia but is unlikely to be sufficient on its own. Critical feminist analysis suggested the need to consider patriarchal influences on men and women in developing change interventions, while social norms theory suggested shifting norms through community and religious leader engagement.
